# Contributions of Socioneuroscience to Research on Coerced and Free Sexual-Affective Desire

**DOI:** 10.3389/fnbeh.2021.814796

**Published:** 2022-01-04

**Authors:** Sandra Racionero-Plaza, Lídia Puigvert, Marta Soler-Gallart, Ramon Flecha

**Affiliations:** Department of Sociology, University of Barcelona, Barcelona, Spain

**Keywords:** socioneuroscience, neurosociology, social sciences, sociology, gender violence, social impact, communicative acts, social interaction

## Abstract

Neuroscience has well evidenced that the environment and, more specifically, social experience, shapes and transforms the architecture and functioning of the brain and even its genes. However, in order to understand how that happens, which types of social interactions lead to different results in brain and behavior, neurosciences require the social sciences. The social sciences have already made important contributions to neuroscience, among which the behaviorist explanations of human learning are prominent and acknowledged by the most well-known neuroscientists today. Yet neurosciences require more inputs from the social sciences to make meaning of new findings about the brain that deal with some of the most profound human questions. However, when we look at the scientific and theoretical production throughout the history of social sciences, a great fragmentation can be observed, having little interdisciplinarity and little connection between what authors in the different disciplines are contributing. This can be well seen in the field of communicative interaction. Nonetheless, this fragmentation has been overcome *via* the theory of communicative acts, which integrates knowledge from language and interaction theories but goes one step further in incorporating other aspects of human communication and the role of context. The theory of communicative acts is very informative to neuroscience, and a central contribution in socioneuroscience that makes possible deepening of our understanding of most pressing social problems, such as free and coerced sexual-affective desire, and achieving social and political impact toward their solution. This manuscript shows that socioneuroscience is an interdisciplinary frontier in which the dialogue between all social sciences and all natural sciences opens up an opportunity to integrate different levels of analysis in several sciences to ultimately achieve social impact regarding the most urgent human problems.

## Neuroscience Asks The Social Sciences

Neuroscience has already demonstrated the influence of social experience and social action on the constitution of the brain (Kentner et al., 2019). Santiago Ramón y Cajal (1989), known as the father of modern neuroscience, already suggested it when he said that each person can, if s/he wants to, be the architect of her/his own brain. The capacity of the brain to change with social experience and action builds upon the hypothesis of brain plasticity, first coined also by Ramón y Cajal (Kandel, 2006), and which is today one of the cornerstones of neuroscience. Brain plasticity refers to the ability of synapses, neurons, and entire brain regions to change their properties in response to use or to different profiles of stimuli (Ramón y Cajal, 1959; Kandel et al., 1991; Kandel, 2006). Eric Kandel, another Nobel Laureate in the field, and one of the most prominent figures in neuroscience today, has also published that “even though I had long been taught that the genes of the brain are the governors of behavior, the absolute masters of our fate, our work showed that, in the brain as in bacteria, genes also are servants of the environment. They are guided by events in the outside world (p. 154)”. Research on twins with identical genes is among the most powerful in showing the influence of experience on the brain, as such studies demonstrate that differences in brain architecture in twin siblings are the result of life trajectories that differ in social experiences and social interactions (Bouchard et al., 1990; Kandel et al., 1991; Kandel, 2006).

Today, there are several studies, particularly in the field of adversity in development, that have proven the ability of social experience to shape the brain. Neuroscientific research (Eluvathingal et al., 2006; Behen et al., 2008) of children raised with little social stimuli and children who are victims of abuse and neglect are particularly enlightening about the negative impact of deprivation of quality social interaction in both brain architecture and brain functioning with subsequent implications that those neural changes can have in deteriorating mental and physical health (Kathryn, 2019; Callaghan et al., 2019). Linking with Kandel’s idea that genes are servants of the environment, studies in epigenetics have proven that the epigenome can be influenced by environmental factors, such as toxic stress derived from violent social experiences, and can end up producing phenotypes and be inherited (Zhang and Meaney, 2010; Hayes, 2018). Along this line, research in animals and humans has shown that traumatic experiences in parents, in particular, emotional trauma, may change their children’s biology (Kaiser, 2014; Curry, 2019). Despite this science still being young, and many questions remain open to exploration, the hypothesis that an individual’s experience might alter the cells and behavior of their children and grandchildren, that our own actions and experiences could biologically affect the lives of our children, puts a very different spin on how we choose to live and also to the decisions that we make throughout life.

Importantly, all this research has evidenced that the ability of the brain to change its properties as a result of environmental stimuli can have two different directions, for the good and for the bad. That is, while some social stressors harm the brain, quality human relationships and quality social experiences benefit the brain. In this sense, longitudinal neuroscientific research on human development, among which the Harvard Adult Development Study stands out, evidences that quality human relationships are better predictors of a healthier, longer, and happier life than genes, IQ or socio-economic status (Waldinger and Schulz, 2010; Harvard Second Generation Study, 2021). That people with better relationships have healthier brains and stronger biological profiles is now well evidenced.

## Some Social Sciences’ Contributions to Neuroscience

In the inquiry into brain plasticity and neural development, neuroscience has embraced contributions from the social sciences. As an example, Eric Kandel, in his seminal book “Principles of Neural Science” (1991) and in “In search of memory” (Kandel, 2006) mentions the behaviorists several times. In particular, he refers to Pavlov, Thorndike, and Skinner and their investigations on reflexive learning as crucial in informing the neuroscientific understanding of implicit memory (Kandel, 2006). Kandel explicitly writes that William James, Thorndike, Pavlov, Skinner, Ulric Neisser, and Freud, who had investigated learning and memory, influenced his work considerably: “Their thinking, and even their errors, provided a wonderfully rich cultural background for my later work” (Kandel, 2006).

The contributions of the social sciences to neuroscience can also be seen in neuroscientific research looking at the effects of adversity, violence, and stress on the brain, and its mitigation. A good example of this is that of the collaboration between the Nobel Laureate Elisabeth Blackburn, and Elissa Epel, a psychologist. Blackburn (1991, 2005) and Armanios and Blackburn (2012) had investigated that poor sleep quality, absence of exercise, unhealthy diet, and even certain chemicals profoundly affect our telomeres, shortening them, meaning an acceleration of the cellular oxidation. This oxidation implies an acceleration of the biological aging of the individual which, in turn, implies an increased likelihood of alterations in immune function and increases in inflammatory markers, which are known to be associated with poorer health outcomes. Yet in looking at the telomere data also from a psychological perspective, attending to socio-economic and socio-cultural factors, but especially to strained and toxic relationships, Blackburn and Epel saw that such relationships produced negative thoughts and chronic stress that also shortened the telomeres (Blackburn and Epel, 2017). Thus, knowledge from clinical and developmental psychology made it possible to make new and complementary meanings of the data, achieving a deeper understanding of telomere functioning and the influence of quality relationships on it *via* the mediation of emotional states.

One more example of contributions of the social sciences to neuroscience is in the field of studies on the neurobiology of attachment (Buchheim et al., 2017). The psychological theory of attachment (Bowlby, 1969, 1982) described attachment as lasting psychological connectedness between human beings (Bowlby, 1969), an emotional bond with another person. Given that humans are social, attachment plays a crucial role in healthy development in any person throughout life. The behaviorists suggested that attachment was a learned process, mostly dependent on feeding. But Bowlby and others proposed that children are born with an innate motivation to form attachments with caregivers. Depending on how social interactions are between babies and caregivers, mostly in terms of responsiveness and availability, different types of attachment emerge, mostly four, with different implications for the social development of the person and her mental health (Lyons-Ruth, 1996; Young et al., 2019). When primary caregivers are available and responsive to the infant’s needs (secure attachment type), then the child acquires a secure base from which to explore the world and other relationships. Contrarily, when secure attachments are not formed early in life, behavior in later childhood and throughout life (Young et al., 2019) can be negatively affected. Neuroscience has embraced this knowledge, for example, in the investigation of the neurobiological implications of impaired early attachment for emotion regulation in abused and neglected children. A famous study in this area is the one on Romanian children raised in institutions during Ceaucescu’s dictatorship in the 1990s, the English and Romanian Adoptees (ERA) study (Sonuga-Barke et al., 2017). These children were abandoned as babies and brutally neglected. Studies employing neuroimage demonstrated that the absence of caring and responsive relationships led to shrunken brains and a number of neural alterations in these children (Sonuga-Barke et al., 2017). Likewise, in light of psychological studies on the positive impact of quality human relationships throughout life (Dunkel Schetter, 2017; Pietromonaco and Collins, 2017), neuroscience has identified that “critical periods may be less restrictive than once thought; in some cases they can be extended or ‘reopened”’ (Kandel et al., 1991), this offering a very transformative view of the brain development of children who have suffered early life adversity (Canzi et al., 2018).

Sociology has also contributed knowledge to current research in cognitive and affective neuroscience. Sociological understandings about primary and secondary socialization (Berger and Luckmann, 1966) have been included in neuroscientific research on the important role that the peer group, and the quality of relationships in it, plays in brain development in adolescence (Telzer et al., 2015). Despite the contributions from the social sciences that the neural sciences have taken into account, they still demand more to make meaning of the many recent and very profound findings on the human brain in relation to social questions such as violence, poverty, racism, etc.

## The Fragmentation of The Social Sciences

Social sciences have made, and are increasingly making, contributions promoting social impact (Aiello et al., 2020) in education (Rios-Gonzalez et al., 2019; Yeste et al., 2019; Duque et al., 2020; Ruiz-Eugenio et al., 2020b), ethnic and cultural minorities (Gómez et al., 2019; Khalfaoui, 2019; Garcia Yeste et al., 2020; Serradell et al., 2020), gender, sexuality and masculinities (Foraster and Morlà, 2019; Serrano Amaya and Ríos González, 2019; Merodio et al., 2020; Salceda et al., 2020), communication and digital media (Madrid et al., 2020; Pulido et al., 2020a, b; Rodríguez et al., 2020) or occupation and organizations (Campos et al., 2020; Mara et al., 2020; Redondo-Sama, 2020; Tellado et al., 2020), among others. However, when we look at the scientific and theoretical production throughout the history of social sciences, a great fragmentation can be observed, having little interdisciplinarity and little connection between what authors in the different disciplines are contributing. This is due to the myth that great production and contributions come from individual authors (Soler-Gallart, 2017).

An example of this fragmentation would be in language and interaction. During the 20th century, very relevant contributions have been made from psychology, especially with the work of Herbert Mead on social psychology in the concept of interaction (Mead, 1934, 1964). He proposed that animals only had conversations by gestures and that it is later in the evolution towards the human being when we move from gestures to symbols or signals, that is, to giving shared meaning to gestures and, hence, these become a symbol. Fire, for instance, is not only an indicator that the forest is burning, but it is also a form of communication through smoke signals between different human beings. Then, as these symbols become more complex, they are perfected and become languages. Hence, overcoming the dualism between the individual and society, Mead argues that the interactions between the individual and society form the individual, that the self is a reflection of those interactions. In his own words, “The ‘I’ is the response of the organism to the attitudes of the others; the ‘me’ is the organized set of attitudes of others which one himself assumes” (Mead, 1934, 175).

Simultaneously, in parallel and without any connection, another social science such as linguistics and specifically linguistic pragmatics, mainly with Austin (1962) and then his pupil Searle (1969), deepens on what language is and how human language works. These authors develop and make contributions to the theory of speech acts, that is, the role that language plays not only in human communication but in constructing social reality itself. Austin distinguishes between locutionary, illocutionary, and perlocutionary acts; to him, the locutionary act is any expression which has meaning, the illocutionary act constitutes the speaker’s intention, and the perlocutionary act would be the action resulting from the act. Searle differs from Austin’s conception by arguing that any act includes the speaker’s intention and, therefore, does not distinguish between locutionary and illocutionary acts. Instead, Searle proposes the distinction between propositional content and illocutionary force, and adds that there is no perlocutionary act but, rather, the perlocutionary effect of a speech act.

On the other hand, and also in parallel and without any connection, in sociology, whose main author Max Weber (1978) already proposed that the animal only has reactive behaviors, we can see a parallelism with Mead’s idea of gestures but without any connection between them. Weber goes on to explain that we go from animals’ reactive behavior to human action, which is characterized by the meaning attributed to it. That is, the animal does not attribute meaning to its behavior, but the human being does, and social or collective actions are those in which several human beings participate and give that action a shared meaning.

The fragmentation of the social sciences cannot be sustained today; in order to provide contributions to science and, most importantly, for those contributions to achieve social impacts, such contributions need to be globalized and in dialogue among the different sciences and disciplines.

## The Contributions of Communicative Acts to Socioneuroscience on Coerced and Free Sexual-Affective Desire

In the 21st century, an unprecedented scientific revolution is taking place, with citizens not only having access to knowledge but also to the co-creation of that knowledge, which overflows the watertight compartments that each science and even each author had been building from their contributions. Today, in order to improve individuals’ lives and societies, we need important research groups and research networks that can contemplate interdisciplinary and global contributions. At this moment, the most advanced point is the dialogic turn that is taking place in societies and in the sciences themselves (Soler-Gallart, 2017; Torras-Gómez et al., 2019). Sciences are becoming increasingly dialogic, not only in establishing interdisciplinarity to contribute from all sciences to the research and creation of knowledge but also in engaging citizens in a co-creation process to produce knowledge (Soler and Gómez, 2020). This ever increasingly dialogic way of conducting science is contributing novel ways in which reality is understood, which in turn entails new ways of researching it.

Within this dialogic shift, the pivotal contribution is the theory of communicative acts (Searle and Soler, 2005; Soler and Flecha, 2010; Soler-Gallart, 2017) developed by the Community of Research on Excellence for All (CREA), which brings together all the contributions of the different social sciences relevant to understand and transform social reality. Yet the development of this theory is not simply a step forward; it is a true Copernican revolution because it globally addresses and gathers all the dimensions and contributions of what we human beings do, which is to communicate with communicative acts. Be it reading a book—as Kandel says, commenting on it, having a sexual relationship, voting, taking a class, participating in a seminar, participating in a musical concert, forming a family, having friendships, etc., all of this is done through communicative acts.

The theory of communicative acts advances and differs from speech acts in four important ways. On the one hand, whereas speech acts only take spoken language into account, communicative acts include any signs of communication in addition to words, such as body language, intonation, gestures, gaze, or context, among others. Second, unlike Austin’s (1962) *illocutionary speech acts* which only include understanding, *illocutionary communicative acts* necessarily include the search for consensus. This understanding also differs from Searle (1969), as for him consensus is part of the *perlocutionary effect*, whereas, in the theory of communicative acts, the *perlocutionary effect* of an *illocutionary communicative act* is what is agreed upon by consensus. Hence, the objective of the *illocutionary communicative act* is not to achieve something, but for the people interacting to construct and achieve consensus, and its *perlocutionary effect* will be whatever they freely agree to do by consensus. Third, *illocutionary communicative acts* require a lack of coercion. However, lack of coercion is not a requirement for *perlocutionary communicative acts*. This means that for a communicative act to be illocutionary, seeking and reaching consensus is not enough; this consensus must be constructed free of coercion. Last, sincerity is not necessarily a requirement for *perlocutionary communicative acts*, but it is for *illocutionary communicative acts*. This does not mean that an action resulting from a *perlocutionary communicative act* cannot be achieved by consensus, or that this consensus cannot be based on sincerity and free of coercion. The difference, then, is that consensus is not a prerequisite for a *perlocutionary communicative act*, as its goal is to lead to action regardless of whether this action is achieved by consensus or not—and even if there is consensus, this might be achieved through lack of sincerity and through coercion. Yet for a communicative act to be *illocutionary*, the goal is to achieve consensus and whatever action results from that consensus, which needs to be constructed based on sincerity and free from coercion.

The theory of communicative acts provides us not only with information on how our human communication works but also on the typology of human communication according to the nature of these acts. This sheds light on which communicative acts favor human values, human rights, the Sustainable Development Goals, the purely human progress, and which communicative acts not only do not collaborate in this progress but harm and attack it, playing a crucial role in society’s and science’s concern for advancing towards the former and overcoming the latter. This framework, thus, allows researchers from all sciences to analyze reality by taking into account all elements of communication, many of which have been overlooked by authors in the social sciences due to the aforementioned fragmentation. By integrating contributions from different sciences, the theory of communicative acts overcomes the imposed speech-body language dichotomy:

The concept of communicative acts enables us to overcome the dualism that opposes speech and body language, intellect and emotions, soul and matter. Communicative acts include all dimensions of people, both what for some is the language of the mind and what for others is the language of the body. Communicative acts include words, tones of voice, looks, caresses, smells, like-nesses, desires, emotions, feelings, etc. They may be separately considered for analytical purposes, but we must always consider they are interrelated in the social reality.

(Soler-Gallart, 2017, 30)

In order to better understand reality and contribute to transforming it, the theory of communicative acts has established a typology of *dialogic communicative acts* and *power communicative acts*. *Dialogic communicative acts* are those based on illocutionary communicative acts and in which dialogic interactions prevail. This does not mean that, even when a male boss and a female employee have a dialogic relationship, one in which both freely share actions, feelings, and desires, there are no power interactions such as the social structure involved. Indeed, in dialogic relationships, there are often power-based interactions, but dialogic ones predominate. On the contrary, *communicative acts of power* are those in which interactions of power prevail, and they include perlocutionary communicative acts aimed at certain actions. This conceptualization of *dialogic communicative acts* goes beyond (Habermas, 1984, 1987) validity claims and becomes more useful when determining when a relationship is based on equality and freedom or on violence. On the one hand, communicative acts not only analyze a person’s claim, but also other elements of the communication such as the social structure in which that claim is said. On the other hand, focusing solely on the claim means only taking into account the speaker’s intention. Yet using and extending Weber’s (1930) ethics of responsibility, the overall consequences of the communicative act—not only of what the speaker says but also, for instance, of the social structure in which it is said—are taken into account. Last, the theory of communicative acts understands dialogue by taking into account both the Apollonian—rationality—and Dyonisian—emotions, feelings, and desires—dimensions, whereas Weber’s validity claims focus only on the Apollonian one.

The different communicative acts, whether they are of power or dialogic, are the ones that neuroscience knows are important for the formation of the brain. What neuroscience has not delved into is what types of communicative acts and interactions constitute one or the other. This is why the contribution of this typology allows neuroscience to study jointly with the human and social sciences how power or dialogic communicative acts influence the brain. In other words, how a power relationship in a couple in which there is psychological or even physical abuse towards a woman or a newborn child influences physical health, mental health, and the brain itself, and how a free, dialogic, satisfactory relationship influences it.

Sexual freedom and consent are increasingly approached by social sciences and discussed in our societies today. We have still not resolved, neither theoretically, nor socially or legally what constitutes consent in a relationship (Flecha et al., 2020). The motto “no means no” has already been substituted by “only yes means yes”, implying that for there to be consent in a sexual relationship, there needs to be an affirmative, conscious and voluntary agreement by all the people involved in the relationship. However, current legal cases in which whether a sexual relationship is based on consent or not is being decided spotlight the need to go beyond words in this analysis. That is, a woman might say “yes” to a sexual relationship, but such a yes might be coerced by the man and/or the environment. In turn, two (or more) people who freely desire to have sex do not necessarily say “yes” to indicate their free desire. Desire might be expressed in different ways, not necessarily through words, and at the same time, words of consent might be expressed with no desire to actually engage in the relationship because the person feels coerced and pressured to do so. The theory of communicative acts, therefore, fills the gap of spoken language by shedding light on the different communicative acts which make a sexual-affective relationship one that is based on consent or on coercion. When analyzing consent—or lack thereof—in a sexual relationship, we need to take into account that, for instance, the context in which the communicative act is occurring might be embedded in institutional power. If a university professor makes a sexual proposal/proposition to a student while the two are revising an exam in the former’s office, the student will not be able to give free consent, even if she provides it, and even if the professor’s sincere aim is to obtain consent. The social hierarchy situates the professor in a power position in which he is in control of the student’s grade and academic success. Following the ethics of responsibility, even if the professor does not intent to coerce the student by using his power to manipulate her grades and only wants to have sex with her if she freely desires so, he cannot ignore the social structures that grant him institutional power over the student, which impedes her from expressing her true desire. Hence, if we analyze the social context of the communicative act, we see that there is coercion even if the professor does not intend to coerce the student.

However, institutional power is not the only element impeding sexual freedom and lack of coercion in a given sexual relationship. The theory of communicative acts also takes into account interactive power, which refers to the power that interactions established among people provide. For example, a woman might agree to have sex with a man, even going to his home without the use of physical force, because if she does not her peers will keep reminding her she is the only virgin in the group or will ask her how long has it been since she last had sex.

These pressures from peers and from men with aggressive attitudes and behaviors are part of the coercive dominant discourse (CDD) that imposes the link between attraction and violent attitudes: “due to imbalanced power relationships between men and women, this coercive dominant discourse (e.g., through TV, teen magazines, social networks, popular media, among other things) influences many girls’ and women’s socialization into linking attractiveness to people with violent attitudes and behaviors” (Puigvert et al., 2019, 2). The theory of communicative acts provides socioneuroscience with a new lens to analyze how the communicative acts that configure the CDD shape the brain and, hence, shape coerced sexual-affective preferences and desires. Through different communicative acts of power, such a discourse forces women, especially adolescents and young girls, into believing that men with aggressive attitudes and behaviors are more exciting and attractive and that egalitarian men are nice but boring and not desirable. The CDD then configures and drives many girls’ preferences and desires (Ruiz-Eugenio et al., 2020a), as not only are they pressured to have relationships (especially hook-ups) with these men, but also to tell their “friends” that they liked doing it, even when they did not (Torras-Gómez et al., 2020). By telling and retelling what they recognize to be a false narrative, they end up assuming such a discourse in their own coerced preferences and desires, becoming part of their own cognitive and affective schemata (Puigvert Mallart et al., 2019). In other words, when a girl hooks up for the first time with a boy with aggressive attitudes and behaviors because her friends tell her it is about time she hooked up with someone, she does not like it, she rejects it and is disgusted by it, she feels it is not what she expected or what her friends told her it would feel like. But because she has assumed the CDD deeply, when telling her friends what it was like, she feels that if she shares her true feelings, her friends will think—and say—she has no idea about sex and will laugh at her. She thus assumes these feelings as her own problem and ends up telling them a different narrative, one in which the boy and the hook-up are portrayed as exciting. Such narrative becomes part of her autobiographical memory, and repeating it over and over again strengthens the synaptic connections that link the boy’s despising and dominating attitudes with pleasure and excitement, normalizing despise and humiliation and finding lack of excitement when someone does not treat them that way (Puigvert Mallart et al., 2019; Ruiz-Eugenio et al., 2020a).

However, the theory of communicative acts also informs socioneuroscience on those dialogic communicative acts that contribute to freed sexual-affective desire. Just as power communicative acts socialize many women in linking attraction to violence, dialogic communicative acts can lead them to a process of re-socialization through dialogic contexts which are critical about the CDD, the narratives it imposes, and the coerced sexual-affective preferences and desires. Interventions that promote dialogic communicative acts to spark reflections about past violent relationships have been found to awaken critical memories. By remembering exactly what they felt during those relationships, by adopting a critical awareness of the way in which the CDD controlled their desire and behavior, women can transform their cognitive and affective schemata and dissociate pleasure from violent attitudes, thus freeing desire (Racionero-Plaza et al., 2020). Only through these dialogic communicative acts can individuals and communities “question and modify the cognitive and affective schemata imposed by the dominant coercive discourse, and thus think, feel, and choose in their intimate life by their own volition, and no by a volition also addicted to the dominant coercive discourse.” (Puigvert Mallart et al., 2019, 212).

## Discussion

From Santiago Ramon y Cajal up to Eric Kandel and other prominent contemporary neuroscientists, neuroscience research has well evidenced that the neural system is greatly influenced and shaped by the social environment. Thus, it is now very clear from neuroscience that the human brain cannot be understood in a vacuum, outside the social world where the person develops. Consequently, it is not only that the human brain facilitates social processes, which is studied by social neuroscience, but also that social experience, social interaction, communication, different types and quality of human relationships, etc., shape the human brain. In turn, those brain changes affect the human experience, including how we feel, who and what is attractive, what is remembered and how, etc., questions that are central in the social problems that individuals and society face. The study of these bidirectional relations between social context and the human brain is the realm of analysis for socioneuroscience. Socioneuroscience takes into account scientific knowledge from all social sciences and all the natural sciences to study the relationships and interlinks between the human brain and human interactions. These interlinks can lead to good or bad directions, depending on which are the social interactions and relationships that individuals are surrounded by and engage in. Research in neuroscience has long had evidence that different interactions and communicative acts lead to very different outcomes. What neuroscience research still lacks is what are the interactions and communicative acts that lead to such different outcomes. This endeavor makes the important role that the social sciences play in neuroscientific studies clear.

Social sciences have made important contributions that have informed neuroscientific research. Kandel (2006), for instance, names several behaviorists as impacting neuroscience’s understanding of implicit memory. Other contributions from social sciences have given neuroscientists a deeper and more nuanced understanding, for instance, of the environment on humans’ telomeres (Blackburn and Epel, 2017) from clinical and developmental psychology, or of the role of peer groups and the quality of relationships in brain development from sociology (Telzer et al., 2015). However, if we look at the social sciences, we see a great fragmentation among the different disciplines and even among authors within the same discipline, following the false idea that relevant contributions come from individual authors. In spite of great contributions to understanding human communication and experience from authors such as Mead, Weber, Austin, or Searle, among others, the fragmentation within their fields and between their works hinders the further potential impact social sciences could have on neuroscience.

In order to respond to society’s increasing demand that science has a clear social impact on the Sustainable Development Goals (SDG), social sciences need to engage in dialogues among themselves, as well as with the natural sciences, in order to deepen into very complex and profound processes that can help individuals and communities improve their lives. Socioneuroscience gathers all these contributions and engages them in dialogue in order to provide theoretical and scientific contributions that will help humanity advance towards its shared goals, such as the SDG. The theory of communicative acts and the empirical research based on it is the pivotal contribution that, as a result of engaging the main works of social sciences, has revolutionized how we study, how we understand, and, most importantly, how we construct and can transform human relationships. Now, we can better understand the development of sexual-affective patterns of attraction, and its neural correlates, that raise the likelihood to engage in violent sexual-affective relationships from a young age; as well as the dialogic and communicative processes that help transform a person’s cognitive and affective schemata. Such a contribution has allowed us to inquire into and understand socialization processes that enslave sexual desire by means of associating it to stimuli related to risk, danger, humiliation, and violence overall (Puigvert Mallart et al., 2019; Racionero-Plaza et al., 2020). This association is imposed on individuals *via* internalization of a coercive dominant discourse (Puigvert et al., 2019) that is sustained through power communicative acts. Socioneuroscience examines the potential translation of this discourse, and its stimulus and response association, into neural circuits in the brain that then coerce the person’s thinking, desire, and behavior, with negative consequences in both the physical and the mental health. However, socioneuroscience has also shed light, taking into account the plastic nature of the human brain, that it is possible for individuals to transform these mental, affective and behavioral patterns *via* new social interactions based on dialogic communicative acts—that is, uniting the Apollonian and Dyonisian dimensions of dialogue.

In all, this manuscript has demonstrated that socioneuroscience is an interdisciplinary frontier in which the dialogue between all social sciences and all natural sciences opens up an opportunity to integrate different levels of analysis in several sciences to ultimately achieve social impact regarding the most urgent human problems.

## Author Contributions

RF and LP conceptualized the contribution. SR-P, LP, MS-G, and RF contributed to the formal analyses and discussion. SR-P wrote the first draft. SR-P, LP, MS-G, and RF revised the manuscript critically for important intellectual content. All authors contributed to the article and approved the submitted version.

## Conflict of Interest

The authors declare that the research was conducted in the absence of any commercial or financial relationships that could be construed as a potential conflict of interest.

## Publisher’s Note

All claims expressed in this article are solely those of the authors and do not necessarily represent those of their affiliated organizations, or those of the publisher, the editors and the reviewers. Any product that may be evaluated in this article, or claim that may be made by its manufacturer, is not guaranteed or endorsed by the publisher.
